# Strain-induced degradation and recovery of flexible NbO_x_-based threshold switching device

**DOI:** 10.1038/s41598-023-43192-w

**Published:** 2023-09-25

**Authors:** Jia Min Ang, Putu Andhita Dananjaya, Calvin Ching Ian Ang, Gerard Joseph Lim, Wen Siang Lew

**Affiliations:** 1 School of Physical and Mathematical Sciences, Nanyang Technological Univcersity, 21 Nanyang Link, Singapore, 637371 Singapore; 2 GLOBALFOUNDRIES Singapore Pte. Ltd., 60 Woodlands Industrial Park D St 2, Singapore, 738406 Singapore

**Keywords:** Electronic devices, Electronic devices, Electronic and spintronic devices

## Abstract

We investigate the functionality of NbO_x_-based selector devices on a flexible substrate. It was observed that the failure mechanism of cyclic tensile strain is from the disruption of atom arrangements, which essentially led to the crack formation of the film. When under cyclic compressive strain, buckling delamination of the film occurs as the compressed films have debonded from their neighboring layers. By implementing an annealing process after the strain-induced degradation, recovery of the device is observed with reduced threshold and hold voltages. The physical mechanism of the device is investigated through Poole–Frenkel mechanism fitting, which provides insights into the switching behavior after mechanical strain and annealing process. The result demonstrates the potential of the NbO_x_ device in flexible electronics applications with a high endurance of up to 10^5^ cycles of cyclic bending strain and the recovery of the device after degradation.

## Introduction

**Figure 1:**
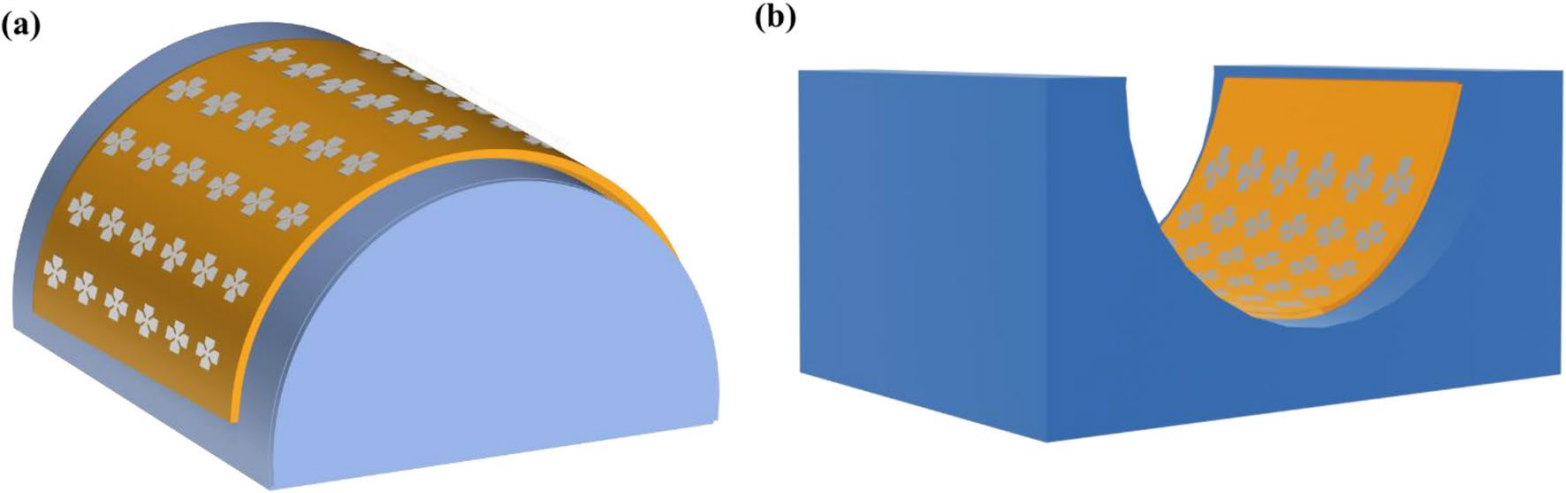
Schematics of polyimide sample mount on 3D-printed mould for in-situ electrical measurements for (**a**) tensile strain and (**b**) compressive strain.

In recent years, flexible electronic devices have garnered huge interest due to their novel applications in many fields ranging from wearable devices, flexible displays, solar cells, sensors, and biomedical devices^1–4^. Unlike conventional silicon wafer-based devices, flexible electronics demonstrate adaptability and conformality to integrate the devices onto applications with curved structures. Furthermore, plastic film flexible substrate has the advantages of being lightweight and low-cost^5^. In different applications, flexible electronic devices are often subjected to different conditions such as deforming, repeated bending, or stretching^6^. Such mechanical strain may cause degradation or failure to the electrical devices due to mechanical reliability issues. Several types of reliability issues may arise depending on the type of mechanical strain performed (Fig. 1). Crack formation tends to occur when tensile strain is applied, whereas buckling and delamination issue tend to occur when compressive strain is applied^7^. For flexible electronic devices to be feasible for practical applications, the device must be able to retain its electrical characteristics when fabricated on a flexible substrate, instead of the conventional silicon wafer substrate. The electrical performance of the flexible electronic device also needs to be consistent when operating under strain.

Over the past decades, there has been an increasing need for a high-performing memory system to support the development of various applications such as embedded systems for the internet of things (IoT) and big data applications, which sees the need for research on flexible memory devices. Hence, more emphasis has been placed on resistive random-access memories (ReRAM) in recent years due to their outstanding memory cell performance. At a lower operating voltage of a few hundred millivolts to a few volts, ReRAM has the advantages of lower power consumption and also excellent scalability, hence rendering it a promising research area for the advancement of the next-generation non-volatile memory^8^. Several studies had demonstrated the potential of flexible ReRAM using materials such as HfO_x_^9^, NiO^10^, TiO_2_^11^, ZrN^12^, and ZnO^13^. However, one major problem in the implementation of ReRAM arrays for high density memory is the sneak path current issue, which degrades the array performance^14^. By integrating a non-linear behavior into the ReRAM device, the sneak path current issue can be reduced as the reading of the on-state current can be done at a higher voltage, while the drop in conductance at the lower voltage regime can seal off the sneak path through the unselected cells^15^. There are a few mitigations on this issue, such as, adopting a one-diode-one-resistor (1D1R) structure^16^, a one-transistor-one-resistor (1T1R) structure^17^, or a one-selector-one-resistor (1S1R) structure^18^. The 1D1R structure is compatible with the unipolar ReRAM structure due to the unipolar nature of the diode devices. However, the bipolar switching behavior is more attractive as it requires a lower operating voltage, a faster switching speed, better uniformity, and a higher endurance performance as compared to the unipolar switching. Though the 1T1R structure is compatible with the bipolar ReRAM, the area of each cell is larger. The selector device, on the other hand, has bipolar switching property and is highly scalable. Hence, the adoption of a 1S1R structure presents a compelling solution to address these challenges and it provides an enhanced performance for flexible electronics applications. However, there has been limited research exploring the performance of a flexible selector device^19–23^ as summarized in Table 1. Such flexible volatile switching devices has been suggested to have promising applications in neuromorphic computing, for instance, a memristor device for the leaky integrate-and-fire (LIF) neuron has been proposed for flexible electronics application^24^. NbO_x_-based selector device is one of such candidates that demonstrates potential as an oscillation neuron for neuromorphic computing applications due to the intrinsic negative differential resistance (NDR) property of such selector devices. This NDR indicates the inherent instability of the device, hence, the IMT devices are able to realize voltage spikes behavior similar to the neurons, which is to amplify and induce the small signals to generate voltage spikes^25^. The possibility of the integration of neuron-like behavior and flexibility within devices such as NbO_x_-based selectors offers a compelling avenue for advancing both neuromorphic computing and flexible electronics.

**Table 1:** Benchmark table of the performance of flexible selector devices.

Selector Structure	Vth (V)	Vhold (V)	Selectivity	Mechanical Endurance	Recovery	Switching Type	Reference
ITO(200)/HfTiO(20)/Pt(200)	1.6	1.1	500	10^3^ Cycles @ tensile strain of 30 mm radius	No	Bi-directional	^19^
Parylene(2000)/Ti(5)/Pt(40)/HfO_2_(20)/Ag(40)/Pt(5)	< 1	–	10^9^	10^3^ Cycles @ strain of 1 mm radius	No	Uni-directional	^20^
ITO(200)/GO(6.8)/HfO_2_(20)/Pt(200)	2.5	1.5	10^3^	10^3^ Cycles @ tensile strain of 20 mm radius	No	Bi-directional	^22^
Ti(10)/Pt(50)/Ag-doped ZnO/Pt(25)	0.5	–	10^7^	10^2^ Cycles @ strain of 15 mm radius	No	Bi-directional	^23^
Ag/OIHP/Ag	0.3	0.18	10^5^	10^4^ Cycles @ strain of 4.7 mm bending radius	No	Bi-directional	^21^
Pt(30)/NbO_x_(20)/Pt(30)	1.7	1.1	> 10^2^	10^3^ Cycles @ tensile strain of 1% or 6 mm radius	Yes	Bi-directional	This Work
				> 10^5^ Cycles @ tensile strain of 0.4% or 15 mm radius			
				10^4^ Cycles @ Compressive strain of 1% or 6 mm radius			

In this work, we reported a flexible NbO_x_-based threshold switching selector device. The effect of mechanical strains on the device had been investigated, and we demonstrate a mechanical endurance of 10^5^ at a lower bending strain of 0.4% and a mechanical endurance of 10^4^ at a bending strain of 1%. It was observed that there is a degradation of the threshold switching device due to the repeated cyclic bending and recovery of the device is possible by implementing an annealing process at 150 °C for 1.5 h. By investigating the conduction mechanism of the devices through Poole–Frenkel mechanism fitting, it is speculated that the effective thickness of the switching layer increases due to the presence of strain-induced cracks. The conduction mechanism further verifies the effect recovery via annealing, as observed through the recovery of effective thickness after annealing. This indicates that the NbO_x_-based device is promising in applications that require flexible electronic devices.

## Results and discussion

### Device electrical characteristics

The electrical performance of the flexible NbO_x_-based threshold switching selector device is first compared to that deposited on the conventional SiO_2_ wafer as the control sample. The flexible NbO_x_ device was fabricated on a Kapton polyimide substrate with an additional 100 nm thick SiO_2_ underlayer to minimize surface roughness. Both devices were characterized by three key electrical parameters obtained via voltage sweeps, which are threshold voltage (V_th_), hold voltage (V_hold_), and off current. As the voltage increases from 0 to 3 V, an abrupt current increase occurs at V_th_ when the device switches to the ON-state or low resistance state (LRS). As the voltage sweeps back from 3 to 0 V, an abrupt decrease in current occurs at V_hold_ when the device switches back to the OFF-state or the high resistance state (HRS). The OFF-state current is defined at the half-write voltage, V_th_/2. All measurements were performed under a compliance current of 1 mA to protect the device from current overshoots and excessive filament formation.

**Figure 2:**
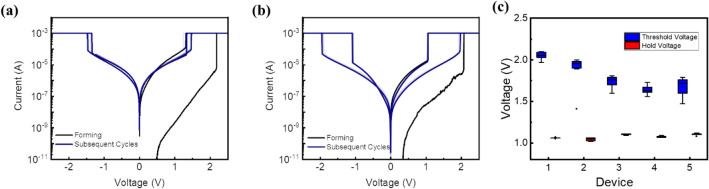
I–V characteristics of Pt(30)/NbO_x_(20)/Pt(30) deposited on (**a**) SiO_2_ wafer and (**b**) polyimide substrate with SiO_2_(100) buffer layer and (**c**) the device to device and cycle to cycle box plot showing the variation in the polyimide sample.

Both pristine devices have a high initial resistance state that require the electroforming process to initiate their switching behaviour. As observed in Fig. 2, the first or forming voltage sweep led to an abrupt current spike corresponding to the electroforming process with V_th_ of 2.08 V and 2.18 V for the polyimide and wafer devices, respectively. As the voltage is swept back to 0 V, the current drops sharply at V_hold_ of 1.07 V and 1.32 V for the polyimide and wafer devices, respectively. While the polyimide device’s V_th_ matches closely to that of the wafer device, the polyimide device’s V_hold_ was slightly lower. The lower V_hold_ for the polyimide sample can be attributed to the lower thermal conductivity of the polyimide material at 0.12 W/m K^26^ as compared to the control sample SiO_2_-based wafers at 148 W/m K^27^. This results in lower heat dissipation from the bottom electrode of the device to the environment, and hence the trapped heat better sustains the filament from rupturing.

Subsequently, both devices' bidirectional threshold switching characteristics were investigated following DC voltage cycles from 0 V → 3 V → 0 V →  − 3 V → 0 V. In these post-forming cycles, both devices showed reliable and repeatable I–V loops, as seen from their closely overlapping plots. Figure 2c also presents the device-to-device and cycle-to-cycle comparison, showing repeatability in device performance. However, the polyimide devices had a significantly larger voltage margin of 0.92 V compared to the wafer device at only 0.18 V. The large discrepancy in voltage margin arose from the much larger decrease in V_th_ post-forming for the wafer device, as V_hold_ of both devices remained comparable to that of the forming cycle. Although both samples demonstrate similar threshold switching behavior, it was observed that the device that is fabricated on the polyimide sample has a lower OFF-state current of 2 µA as compared to the control sample device with an OFF-current of 10 µA.

Additionally, the devices show a difference in behaviour when turning off under a decreasing voltage sweep. The SiO_2_ sample showed an abrupt increase in resistance back to its HRS at V_hold_, while the polyimide sample showed an abrupt increase to a lower resistance state that only gradually approaches its HRS with decreasing voltage, as seen in the gapped and non-overlapping I–V curves under opposite voltage sweeps in Fig. 2b. This suggests that the rupturing of the filament for the polyimide sample was not as thorough as the control sample at the hold voltage. Instead, there will be some residual metallic filament due to the lower heat dissipation from the device to the environment as the thermal conductivity of the polyimide material is lower.

### Electrical characteristics under tensile strain

The robustness of the device performance against tensile strain was demonstrated on custom 3D-printed molds in this section. The bending strain can be expressed in the following equation ^28^:

1 $$\begin{array}{*{20}c} {{\upvarepsilon }_{M} = \frac{h}{2r},} \\ \end{array}$$

where ${\upvarepsilon }_{M}$ is the bending strain, *h* is the thickness of the substrate and layers, and *r* is the bending radius applied to the substrate.

Each mold, as shown in Fig. 1, was designed following Eq. (1), where the thickness of the polyimide substrate is 120 µm, to apply tensile strains ranging from 0.3% to 1.0% along the top electrode. The I–V switching characteristics were measured following the sequence of pre-strain (0%), increasing in-situ strain (0.3%, 0.4%, 0.6%, 0.75%, and 1%), and the flattened post-strain (0% PS) states. At each state, 10 I–V switching characterization were performed. The strain was applied in the top electrode direction.

**Figure 3:**
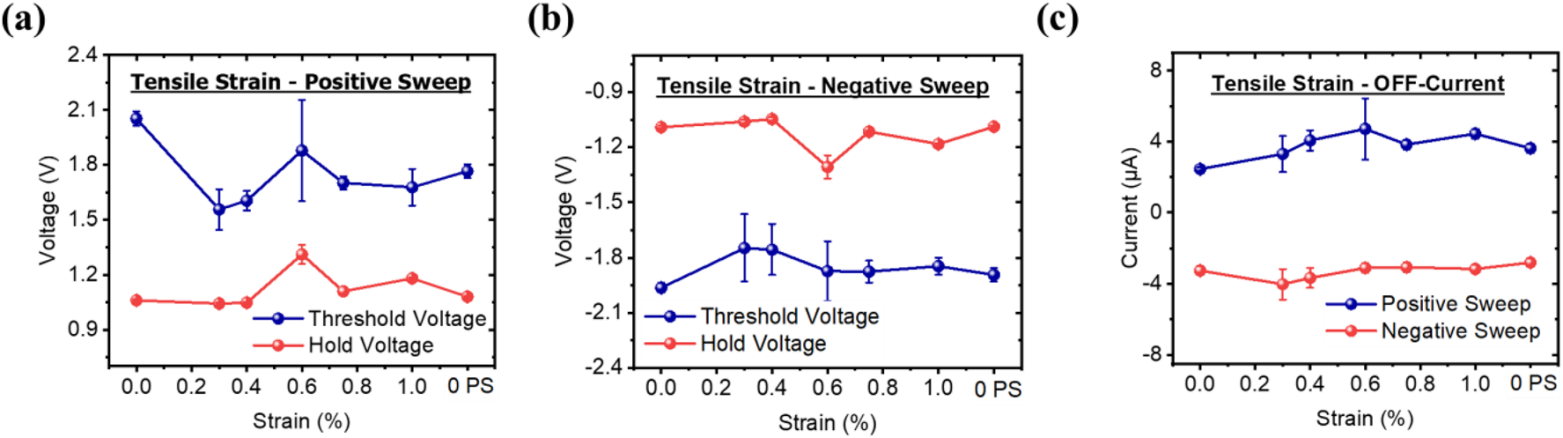
Pre-strain, in-situ strain, and post-strain electrical characteristics of the (**a**) threshold voltage and hold voltage in the positive sweep, (**b**) threshold voltage and hold voltage of the negative sweep, and (**c**) the off-current of both positive and negative sweep for device under tensile strain.

Figure 3 presents the results of the measurement of the threshold voltage, hold voltage, and the OFF-state current that was obtained for every cycle. The device exhibits bidirectional switching with symmetrical switching behavior for both the positive and negative voltage sweeps. The average value of the threshold voltage and the hold voltage is at 1.75 V and 1.12 V, respectively, with a coefficient of variation (CV) of 11% and 8% for the positive sweep (Fig. 3a). For the negative sweep, the average value of the threshold voltage and the hold voltage is at − 1.85 V and − 1.13 V with a CV of 6.92% and 7.82% for the negative sweep (Fig. 3b), respectively. The OFF-state current, on the other hand, shows an average value of 3.75 µA with a higher CV value of 28% for the positive sweep, and 3.28 µA and 17% for the negative sweep (Fig. 3c). An average selectivity of > 10^2^ was observed for the device measured. The measured values indicate that higher fluctuations in the threshold voltage, hold voltage, and the OFF-state current was observed when the device is under strain of 0.2% to 1% as compared to when the device is not strained. However, the variations are arbitrary and do not impact the device performance.

### Electrical characteristics under compressive strain

**Figure 4:**
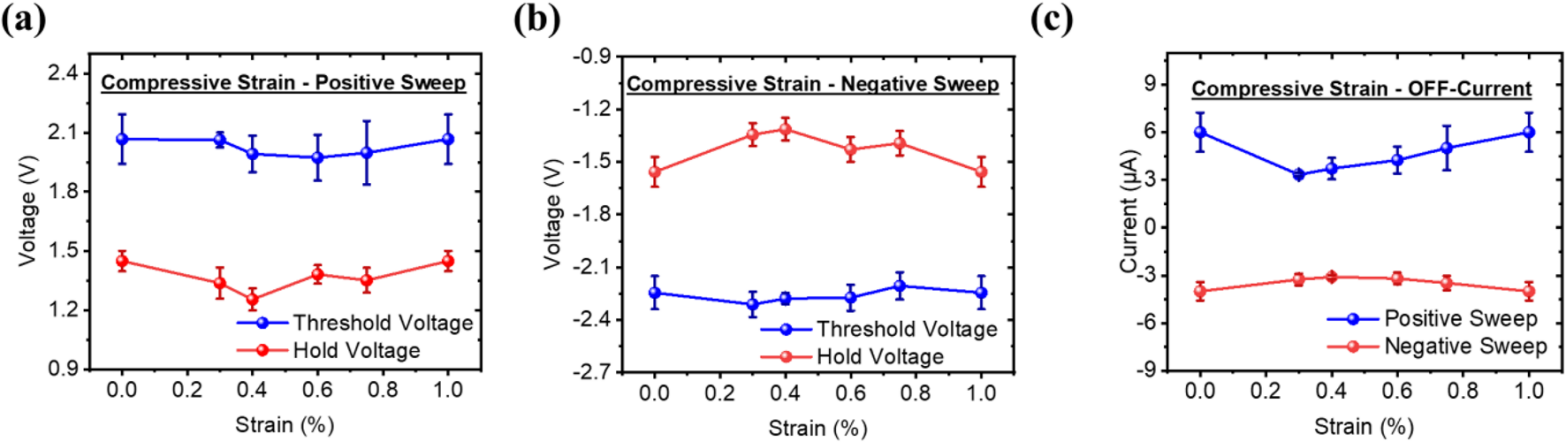
Pre-strain, in-situ strain, and post-strain electrical characteristics of the (**a**) threshold voltage and hold voltage in the positive sweep, (**b**) threshold voltage and hold voltage of the negative sweep, and (**c**) the off-current of both positive and negative sweep for device under compressive strain.

The effect of compressive strain on the NbO_x_-based devices was investigated in this section, and an even better degree of device robustness was observed. The sequence of strain measurements was performed, as described in Section "Electrical characteristics under tensile strain", but on concave molds. Figure 4 presents the results of the measurement of the threshold voltage, hold voltage, and the OFF-state current that was obtained for every cycle. Similar to the device under tensile strain, the device exhibits bidirectional switching with symmetrical switching behavior. The average threshold voltage and the average hold voltage are at 1.75 V and 1.12 V respectively for the positive sweep, and − 1.85 V and − 1.13 V for the negative sweep. The device under compressive strain exhibits less voltage fluctuation when compare with the device under tensile strain with the CV of the sweeps being 5.85%, 6.47%, 3.58%, and 8.36%, respectively. The OFF-state current, however, demonstrates similar fluctuations with an average value of 4.72 µA and a CV value of 30% for the positive sweep, and 3.5 µA and 16% for the negative sweep. These fluctuations were observed with the applied compressive strains, but they did not significantly impact the device's performance.

### Mechanical endurance under tensile strain

**Figure 5:**
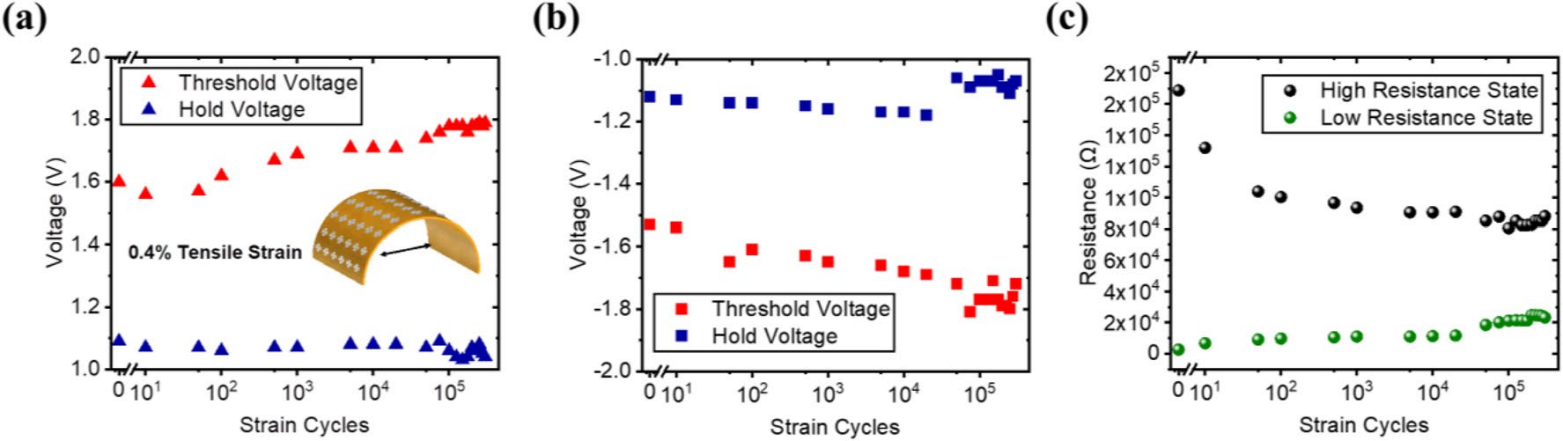
Mechanical tensile endurance at 0.4% strain showing (**a**) cycle-to-cycle voltage for the positive sweep, (**b**) cycle-to-cycle voltage for the negative sweep, and (**c**) cycle-to-cycle resistance state.

The mechanical endurance of the device under tensile strain was investigated in this section. Repeated cyclic bending of the substrate was performed via a homebuilt computer-controlled cyclic bending equipment. The I–V characteristics were measured after multiple strain cycles, and the process was repeated for > 10^5^ cycles at 0.4% strain (Fig. 5). As the number of strain cycles applied on the substrate increases, the voltage margin of the device broadens with a gradual increase in the threshold voltage and a slight decrease in hold voltage for both the positive sweep and the negative sweep (Fig. 5a,b). The selectivity, on the other hand, decreases sharply after the first 10 bending cycles and shows a continual gradual decrease as the strain cycle increases (Fig. 5c).

**Figure 6:**
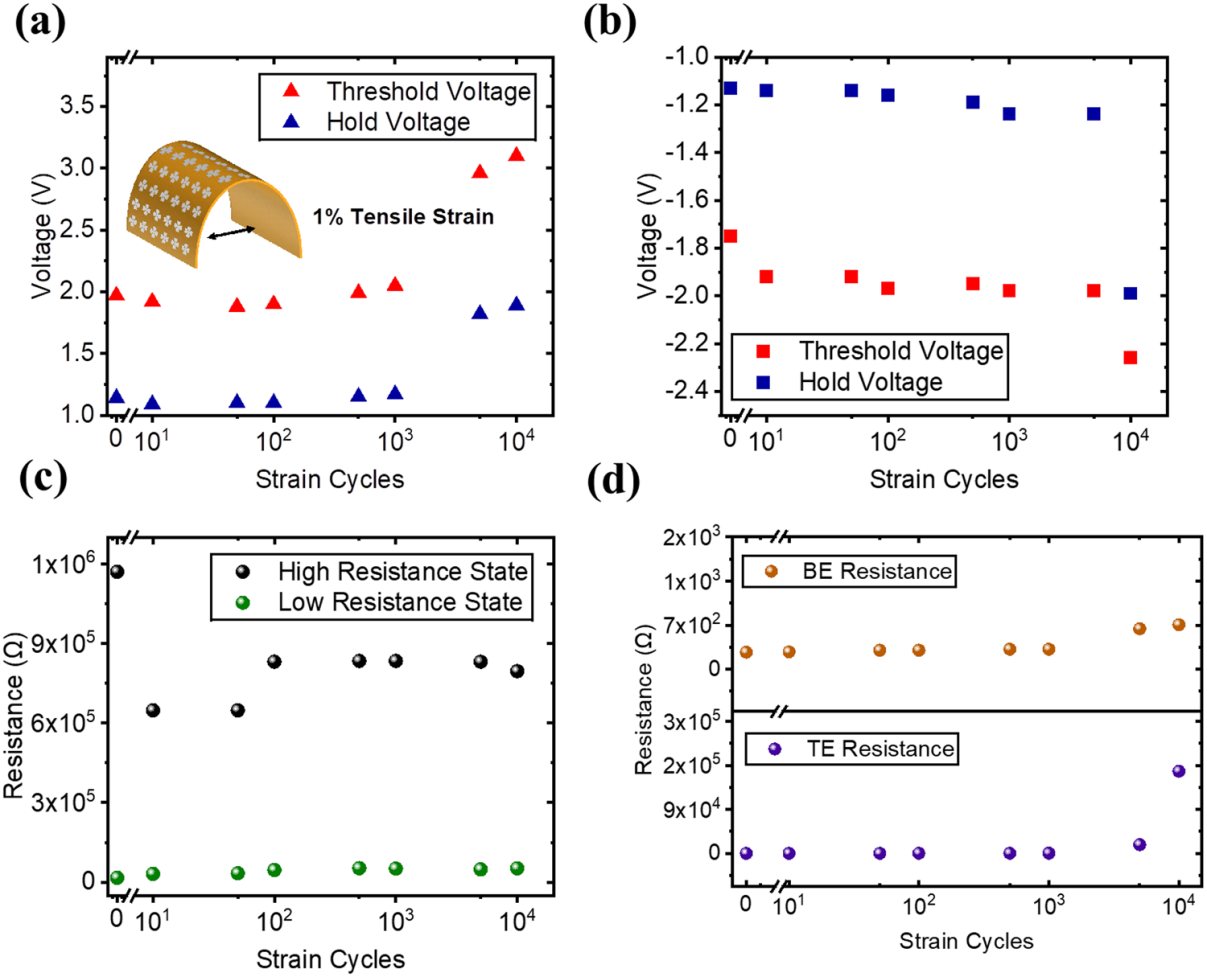
Mechanical tensile endurance at 1% strain showing (**a**) cycle-to-cycle voltage for the positive sweep, (**b**) cycle-to-cycle voltage for the negative sweep, (**c**) cycle-to-cycle resistance state, and (**d**) the resistance of the top electrode (TE) and the bottom electrode (BE).

The mechanical endurance under tensile strain was further investigated at a higher strain of 1% (Fig. 6). The threshold voltage and the hold voltage of the device remain stable for the first 10^3^ cycles, and signs of degradation can be observed thereafter with a huge increase in both the threshold and hold voltages (Fig. 6a,b). The selectivity, however, shows the same trend as when under a smaller 0.4% strain whereby it decreases sharply after the first 10 bending cycles. After the first instance of bending, fluctuations were observed but the selectivity remains relatively stable.

The degradation of the device was further investigated by the resistance value of the electrodes after the bending cycles (Fig. 6d). Prior to bending, the BE resistance was measured to be 282 Ω which then increased to 738 Ω after 10^4^ bending cycles. The TE resistance, on the other hand, shows a huge increase from 255 Ω to 174 kΩ after the cyclic bending. Under repeated strain, disruption of the atom arrangement occurs, and such movement of dislocations tends to pile up against the grain boundary^29^. Eventually, the extrusions and intrusions contribute to the crack formation on the film. Due to the ductility of the Pt film^30^, the cracks were formed as micro-cracks which degrades the performance of the device but remained functional.

**Figure 7:**
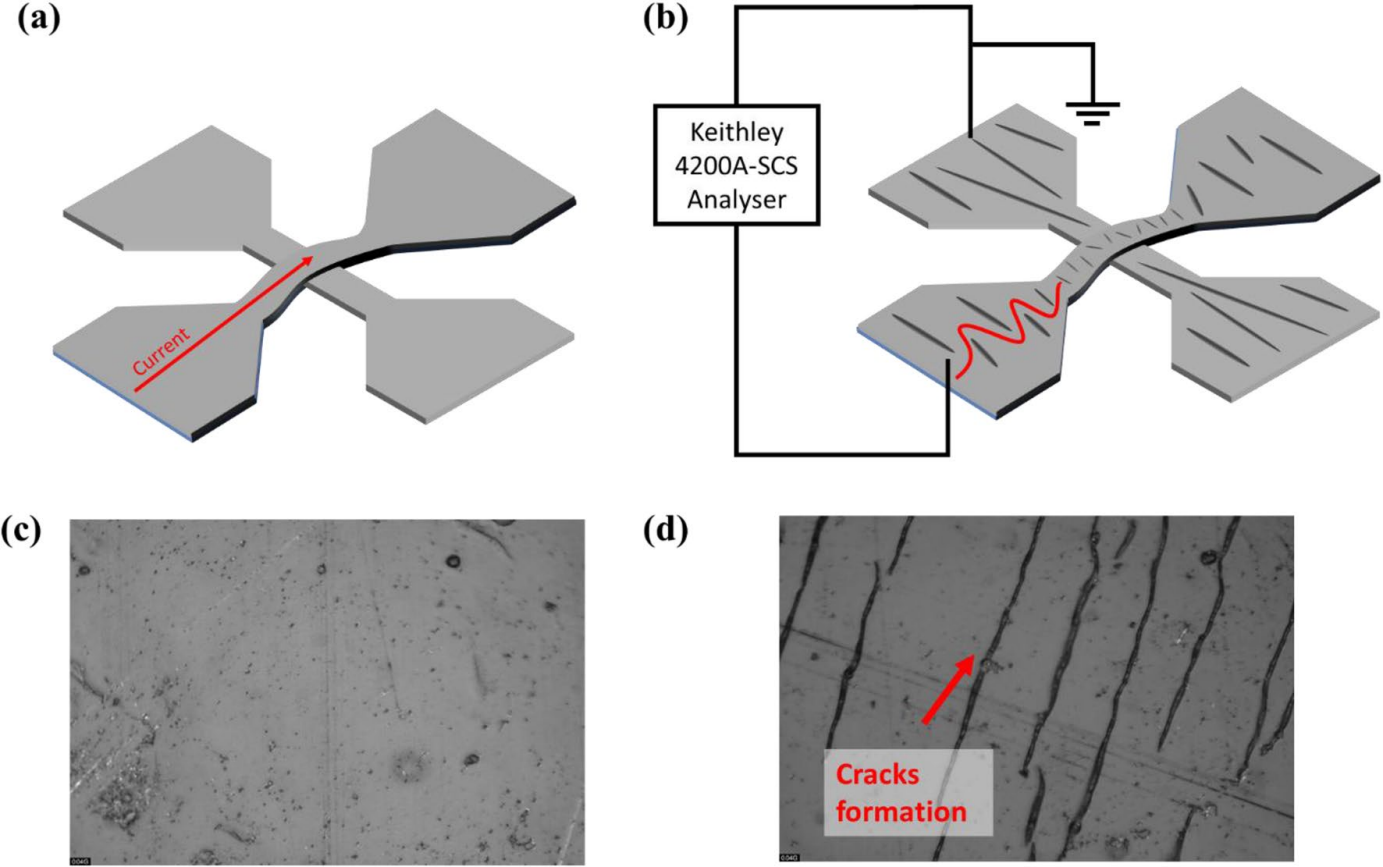
Schematics of the current path of the device (**a**) without cracks and (**b**) with micro-cracks formation.

Micro-cracks were formed perpendicular to the strain direction which obstructs and lengthen the current path through the TE, hence increasing the resistance of the TE after 10^4^ cycles (Fig. 7). On the other hand, the direction of the current flow is parallel to the BE, hence the increase of the resistance after bending is not as significant as compared to the TE resistance change.

### Mechanical endurance under compressive strain

**Figure 8:**
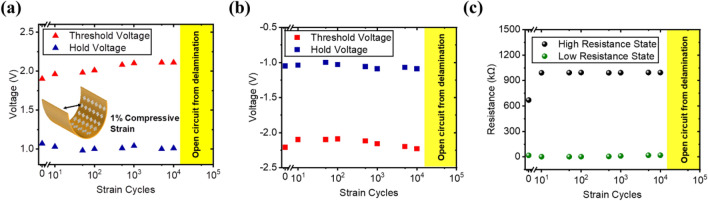
Mechanical compressive endurance at 1% strain showing (**a**) cycle-to-cycle voltage for the positive sweep, (**b**) cycle-to-cycle voltage for the negative sweep, and (**c**) cycle-to-cycle resistance state.

The mechanical endurance of the device under compressive strain was investigated in this section. The cyclic bending was performed at a compressive strain of 1%, and the device exhibits a more stable switching behavior compared to when under tensile strain. For the positive sweep, the average threshold voltage and hold voltage is at 2.03 V and 1.02 V, with a variation of 3.9% and 2.7% respectively (Fig. 8a). As for the negative sweep, the average threshold voltage and hold voltage is at − 2.15 V and − 1.05 V, with variations of 2.6% and 2.9% respectively (Fig. 8b). The selectivity of the device increases slightly after the first 10 bending cycles and exhibits stable resistive states thereafter (Fig. 8c). The device under compressive strain demonstrates stable switching behavior of 10^4^ strain cycles. However, the probe pads of the device were disconnected after repeated cyclic bending. This is due to the buckling delamination of the film which occurs when the compressed films have debonded from their neighboring layers, which is a common failure mechanism of compressive strain^31^.

### Recovery of strain-induced degradation via annealing

The discussions in Section "Mechanical endurance under tensile strain" observed that the repeated tensile strain of 1% causes the degradation of the device in which there is an increase in the threshold voltage and hold voltage. Correspondingly, the resistance of the top electrode increases due to the direction of strain applied to be parallel to the top electrode, which causes cracks that are perpendicular to the top electrode. At different annealing temperatures, there are different stages of structural changes including recovery, recrystallization, and grain growth^32^. The recovery stage is no longer possible when the annealing temperature is too low. On the other hand, annealing at excessively high temperatures can introduce a new set of challenges, including the potential for irreversible damage to the NbO_2_ devices. As the polyimide Kapton substrate is not resistant to high temperatures, an optimum annealing temperature of 150 °C was chosen to balance between promoting effective recovery mechanisms and irreversible thermal effects on the device components. This temperature facilitates the movement of dislocations through thermally-assisted depinning, as well as the subsequent re-arrangement of dislocations, which contribute to strain relief and enhanced device stability, while avoiding irreversible thermal damage to the devices and the substrate material.

**Figure 9:**
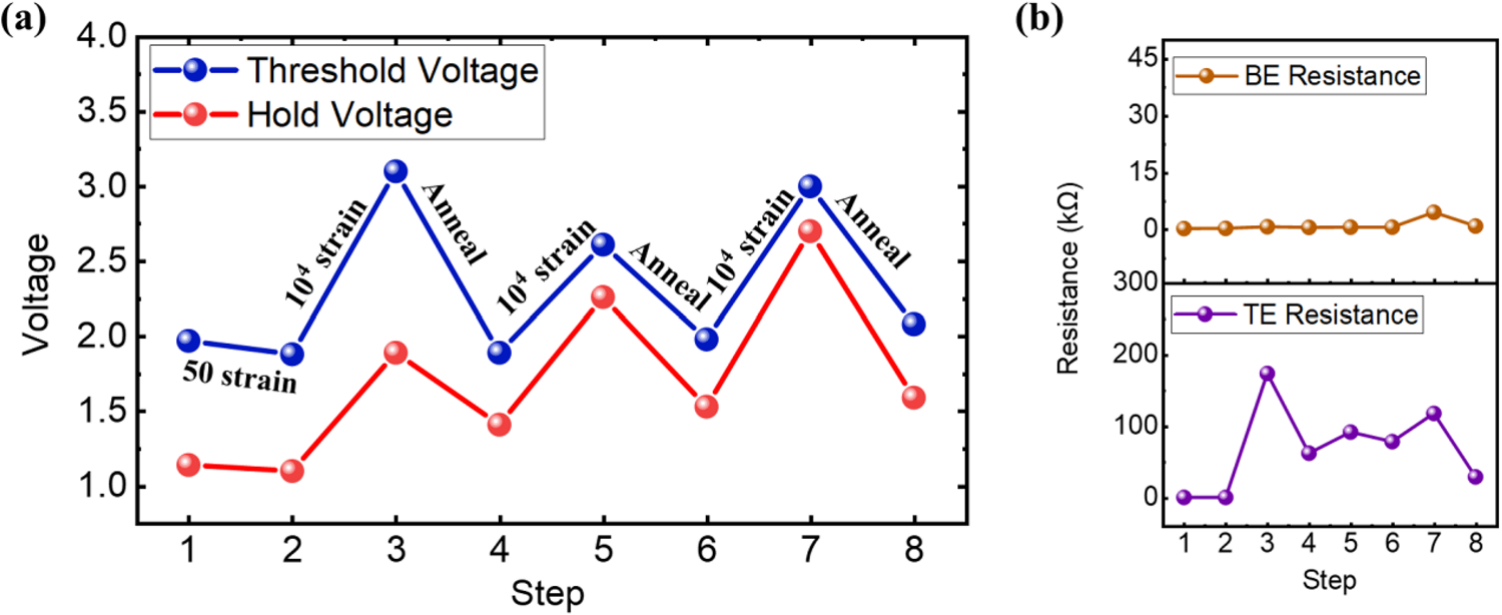
(**a**) Threshold voltage and hold voltage of device after repeated straining and annealing steps and (**b**) the corresponding resistance of the electrodes at each step. The annealing steps were performed at 150 °C.

By subjecting the device to an annealing treatment at 150 °C for 1.5 h, the device is able to recover its switching behavior with the reduction of the threshold voltage and hold voltage, as shown in Fig. 9. The strain recovery effect of the device was observed, which can be attributed to the reduction or re-arrangement of the defects in the crystal lattice, or the movement or re-arrangement of the dislocations into the lower energy configuration^33,34^. This aids in the relieving of the internal strain energy, which leads to restoring the performance of the device. The annealing was performed in vacuum condition (5E-8 Torr) instead of atmospheric condition to prevent the oxidation of the NbO_x_ to Nb_2_O^35^. The measured XPS spectra that shows the unaffected NbO_x_ stoichiometry after the strain and annealing cycle is included in the supplementary information as Figure S1. The stress fatigue induced by the repeated strain causes an increase in crack density, which eventually results into long-range cracks that obstruct the current flow^36^. Hence, the resistance of the top electrode was increased drastically from 255 Ω to 174 kΩ at the first instance of 10^4^ bending cycles (Fig. 9 step 2–3). However, the resistance was reduced to 62 kΩ via the annealing treatment from step 3 to step 4 in Fig. 9.

Subsequent steps show further strain-induced degradation after 10^4^ cycles and recovery of the device was repeatable.

**Figure 10:**
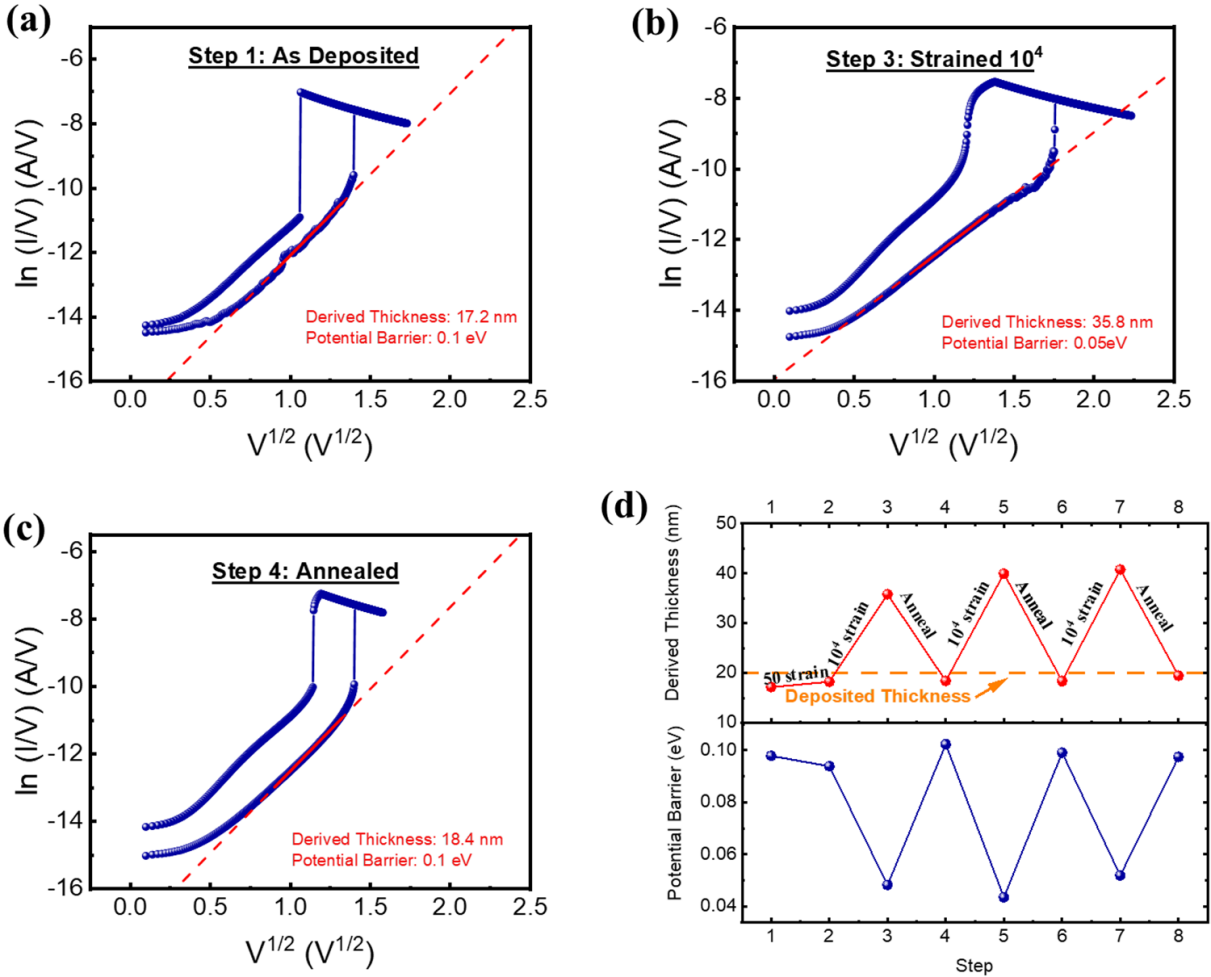
P-F fitting on $ln(\frac{I}{V})$ vs $\sqrt{V}$ plot at (**a**) as-deposited step, (**b**) step after 10^4^ strain cycles, (**c**) after annealing step, and (**d**) the extracted thickness and potential barrier of the various steps.

The conduction mechanism of the device was further investigated to study the underlying physical mechanisms at the various straining and annealing steps. The Poole–Frankel (P–F) emission is one of the bulk-limited conduction mechanism which can be expressed as

2 $$J_{PF} = {\text{q}}\mu N_{C} {\text{Eexp}}\left[ {\frac{{ - {\text{q}}\left( {\Phi_{T} - \sqrt {{\text{qE}}/\pi \varepsilon } } \right)}}{{{\text{kT}}}}} \right]$$

with the constant in the equation being defined in table S1 in the supplementary information^37^. The experimental data was analysed to investigate the conduction mechanism in the high resistance state and high voltage regime, as it is anticipated that the P-F mechanism is a field-assisted emission mechanism which is more likely to occur in the high field regime. By performing a linear fit on the graph $ln(\frac{I}{V})$ vs $\sqrt{V}$, the derivation of the switching layer thickness and the potential barrier was obtained. Prior to any strain and annealing step (Fig. 9a step 1), the derived potential barrier based on the P-F mechanism is ${\Phi }_{B}=0.1eV$ and the derived thickness is 17.2 nm (Fig. 10a). The obtained potential barrier is in accordance with the values which that the effect of P-F emission causes the potential barrier to be lowered from 0.34 eV to 0.1 eV^38^. After the mechanical tensile strain of 10^4^ cycles (Fig. 9a step 3), the derived thickness increases to 34.8 nm whereas the potential barrier decreases to 0.05 eV (Fig. 10b). It can be speculated that the mechanical strain causes micro-cracks within the NbO_x_ layer, hence increasing the effective switching path/thickness of the switching layer. The annealing process allows for the recovery of the device through the rearrangement of dislocations and relieve from residual stresses on the substrate and thin film layers^39,40^ (Fig. 9a step 4), and this is also observable through the conduction mechanism fitting. The derived thickness and potential barrier reverted back to a value of 18.4 nm and 0.1 eV respectively (Fig. 10c). Further verification of the conduction mechanism for the subsequent strain and recovery steps (Figure S2 in the supplementary information) reveals similar behavior in which the thickness and potential barrier is derived and summarized in Fig. 10d.

## Conclusion

In this work, we demonstrated the functionality of a NbO_x_-based selector device on a flexible substrate. The effect of various strain-induced degradations had been investigated – repeated bending stress at a lower tensile strain of 0.4%, repeated bending stress at a higher tensile strain of 1%, and repeated bending strain at a compressive strain of 1%. It was observed that the failure mechanism differs for the different types of strain in which for tensile strain at 0.4%, the gradual degradation of the device occurs with the narrowing of the resistance states and the increasing of the threshold voltage. At 1% tensile strain, the degradation occurs abruptly after 10^3^ cycles and there is a huge increase in both the threshold and hold voltages due to crack nucleation. As for the compressive strain, the electrical performance of the device was stable for 10^4^ cycles, but the failure mechanism is related to the buckling delamination of the film. The physical mechanism had been verified using Poole–Frenkel mechanism, which shows that the mechanical strain results in a longer effective path/thickness of the switching layer. By implementing an annealing process after the strain-induced degradation, recovery of the device is observed with reduced threshold and hold voltages as well as the reverting of the effective thickness observed in the P-F mechanism fitting. The result demonstrates the potential of the NbO_x_ device in flexible electronics applications with a high endurance of up to 10^5^ cycles of cyclic bending strain and the recovery of the device after degradation.

## Methods

For the NbO_x_-based devices, a metal–insulator-metal (MIM) crossbar structure was employed. A 100 nm thick SiO_2_ buffer layer was deposited onto the Kapton polyimide substrate via plasma-enhanced chemical vapor deposition (PECVD) prior to the fabrication of the device. Magnetron sputtering was used for all subsequent sputtering processes, and argon plasma treatment was implemented prior to deposition processes to reduce possible surface contaminants. Both the top electrode and the bottom electrode are 30 nm thick Pt layers, in order to achieve symmetrical structure and device performance at opposite polarities. The insulating layer is a 20 nm thick NbO_x_ layer that was deposited through reactive sputtering of a metallic Nb target. The reactive sputtering was performed at an oxygen flow rate of 3 sccm and an argon flow rate of 20 sccm. UV lithography was used for the patterning of the structures with a device size of 5 µm by 5 µm.

**Figure 11:**
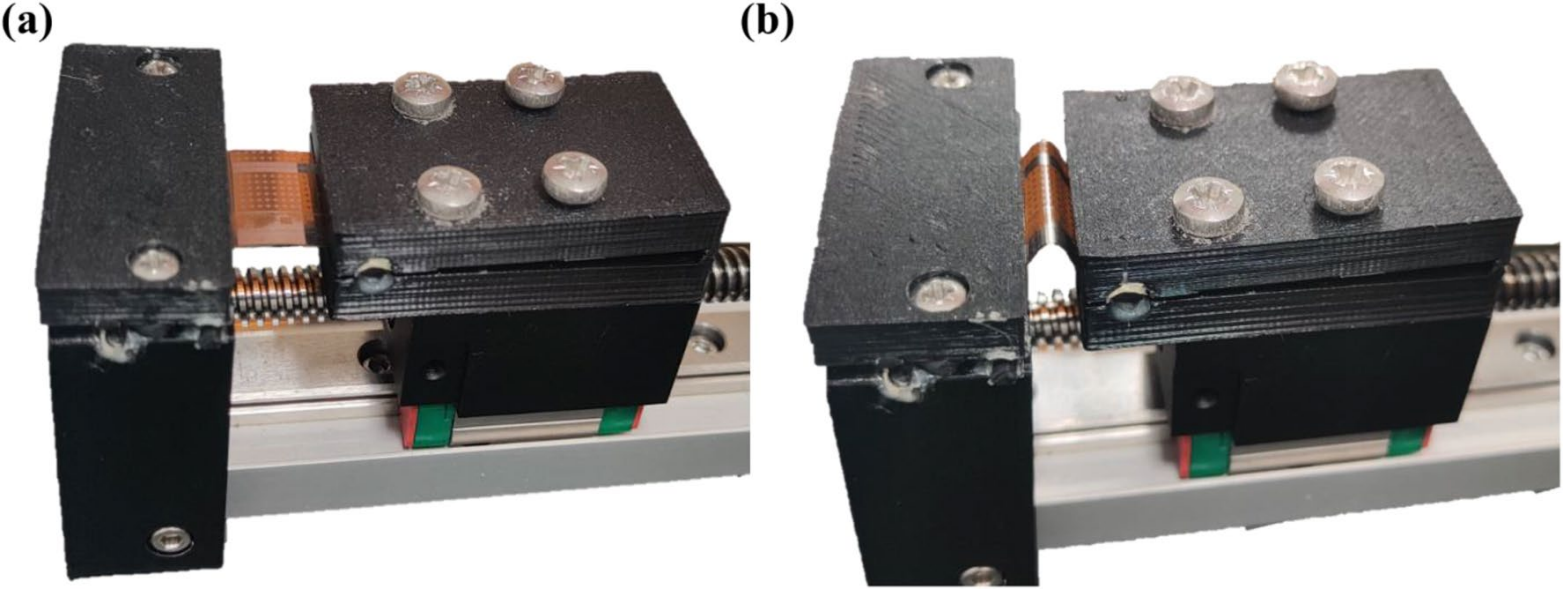
Cyclic bending machine clamping the polyimide substrate (**a**) without strain and (**b**) under strain.

All electrical characterization was measured via the Keithley 4200A-SCS semiconductor parameter analyzer. *In-situ* electrical measurement was performed by mounting the flexible substrate onto 3D-printed molds of different bending radii. The mechanical endurance was performed via a home-built computer-controlled cyclic bending machine using a stepper motor coupled with a microcontroller (Fig. 11).

### Supplementary Information

Supplementary Information.

## Data Availability

The datasets used and/or analysed during the current study are available from the corresponding author on reasonable request.
