# Supplementary material for: Strain-induced degradation and recovery of flexible NbOx-based threshold switching device
Source: Sci Rep. 2023 Sep 25;13:16000. doi: 10.1038/s41598-023-43192-w (PMC10519953; doi:10.1038/s41598-023-43192-w)

**Supplementary Information**

**Figure S1.** XPS spectra of (a) O deconvoluted fitting for the as-deposited sample, (b) Nb 3d deconvoluted fitting for the as-deposited sample, (c) O deconvoluted fitting and (d) Nb 3d deconvoluted fitting for the sample after multiple strain & annealing cycles.


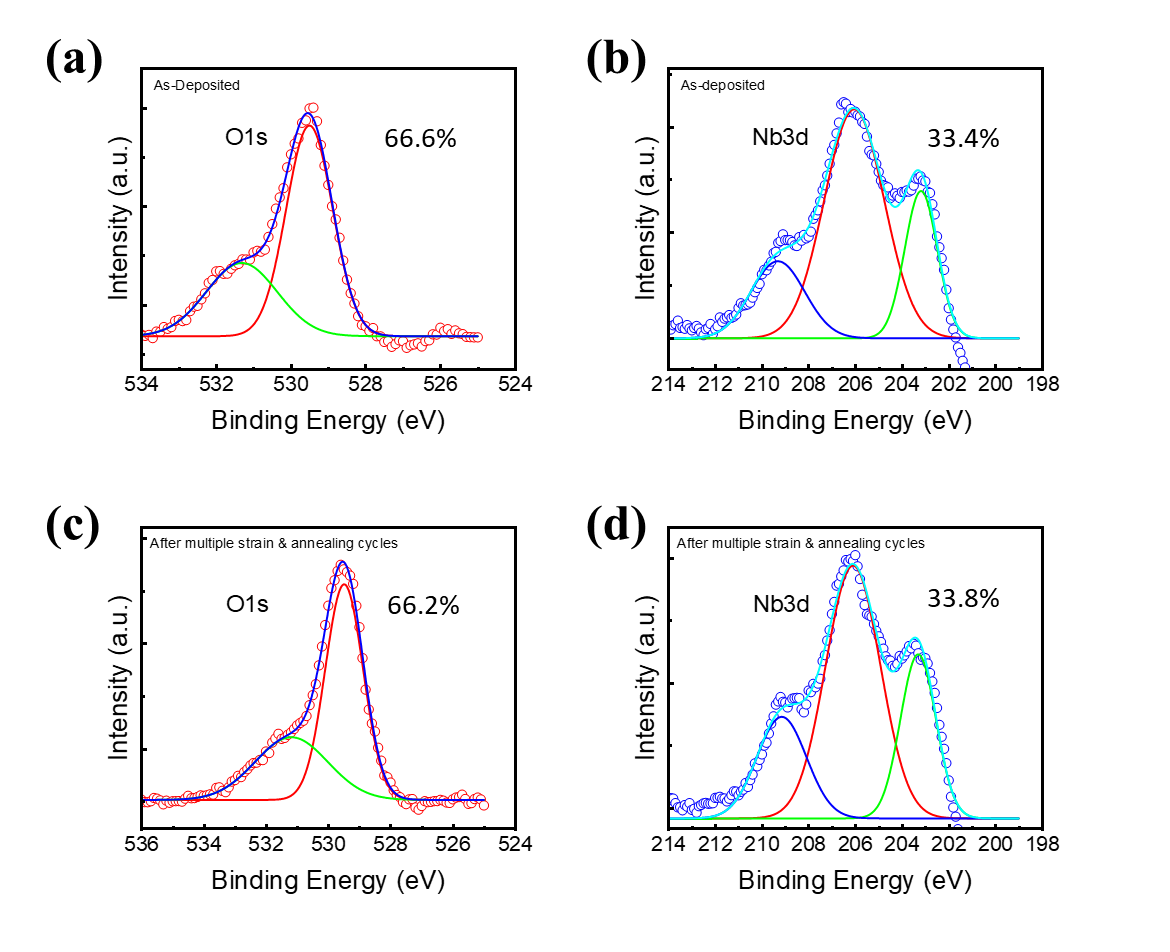


**Table S1.** Fitting parameter and constant value of NbO_x_^.^

| **PARAMETER** | **NAME** | **NbO_x_ Value** | **UNIT** |
| --- | --- | --- | --- |
| $q$ | Elementary charge | $1.60218\times{10}^{-19}$ | C |
| $\varepsilon_{0}$ | Permittivity of free space | $8.85419\times{10}^{-12}$ | $Fm^{-1}$ |
| $\varepsilon_{r}$ | Relative permittivity | 20 |  |
| $k$ | Boltzmann constant | $1.38\times{10}^{-23}$ | $eVK^{-1}$ |
| $T$ | Temperature | 298.15 | $K$ |
| $\mu$ | Electronic drift mobility | 0.003 | $cm^{2}V^{-1}s^{-1}$ |
| $N_{c}$ | D OS in conduction band | $5.6\times{10}^{19}$ | $cm^{-3}$ |

**Figure S2.** P-F fitting on $ln(\frac{I}{V})$ vs $\sqrt{V}$plot at subsequent (a) 10^4^ strain cycles, (b) annealing step, and (c) 10^4^ strain cycles, and (d) final annealing step.


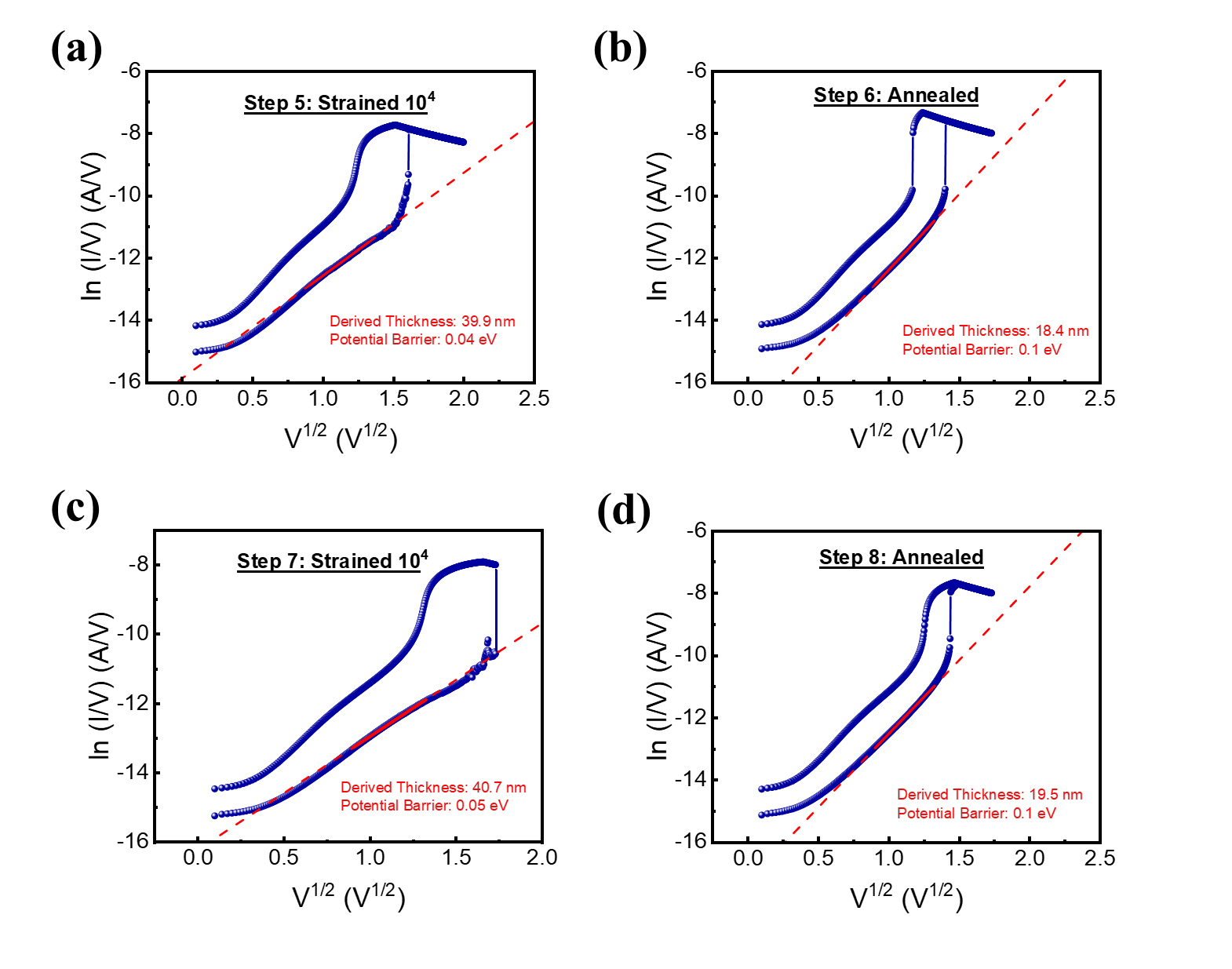

Supplement: Supplementary file 1 — Supplementary Information. [file 41598_2023_43192_MOESM1_ESM.docx]
